# ICTV Virus Taxonomy Profile: *Bicaudaviridae*

**DOI:** 10.1099/jgv.0.001106

**Published:** 2018-06-07

**Authors:** David Prangishvili, Mart Krupovic, ICTV Report Consortium

**Affiliations:** Department of Microbiology, Institut Pasteur, 25 rue du Dr. Roux, 75015 Paris, France

**Keywords:** *Bicaudaviridae*, ICTV Report, taxonomy, Acidianus two-tailed virus

## Abstract

The family *Bicaudaviridae* includes viruses that infect hyperthermophilic archaea in the genus *Acidianus*. The circular double-stranded DNA genome of Acidianus two-tailed virus consists of 62 730 bp, and replication can be either lytic or lysogenic. Virions undergo unique extracellular morphogenesis, being released from host cells as spindle-shaped particles that subsequently develop long tails, one at each of the two pointed ends. The spindle-shaped morphology represents a group of archaea-specific virion morphotypes. This is a summary of the International Committee on Taxonomy of Viruses (ICTV) Report on the taxonomy of the *Bicaudaviridae* which is available at www.ictv.global/report/bicaudaviridae.

## Virion

**Fig. 1.**
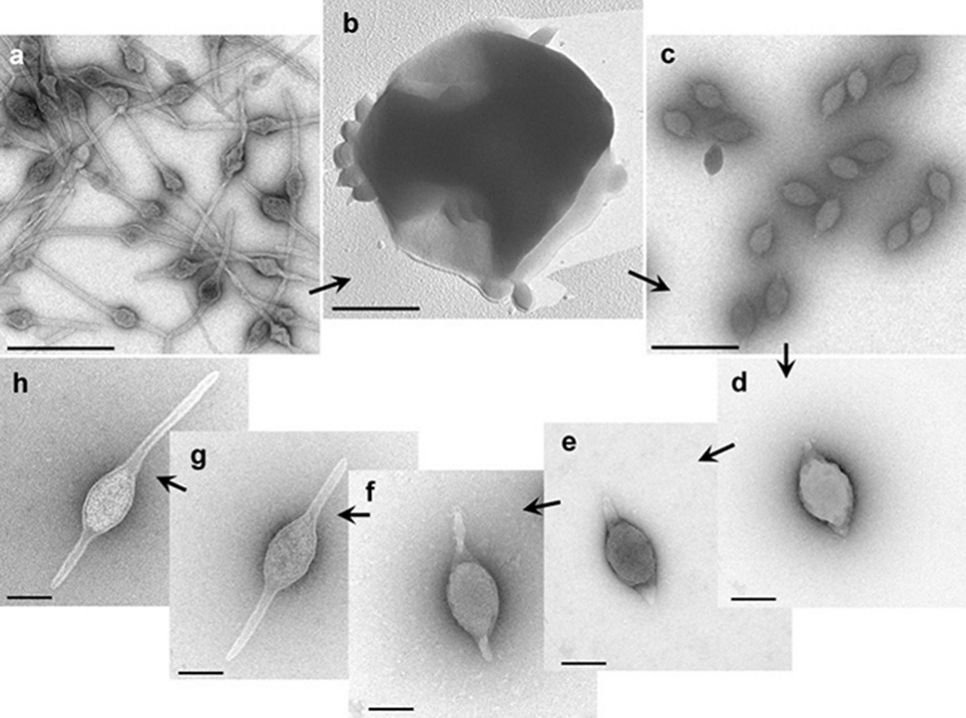
Electron micrographs of different forms of Acidianus two-tailed virus. Virions a, in infected cell culture at late stage of tail development. b, extruded from an *Acidianus convivator* cell; c, in growing culture of infected *A. convivator*, 2 days post-infection; d, as for c, but purified by CsCl gradient centrifugation; e–h, as for d, but after incubation at 75 °C for 2, 5, 6, and 7 days, respectively. Samples were negatively-stained with 3 % uranyl acetate, except for b, which was platinum-shadowed. Bars, a–c, 0.5 µm; d–h, 0.1 µm. (First published in [2]).

Virions are released from host cells as spindle-shaped particles, about 120×80 nm, which subsequently develop two long tails, one at each of the two pointed ends (Table 1, Fig. 1) [1, 2]. The tails are heterogeneous in length, reaching 400 nm, consisting of a tube-like structure with a wall approximately 6 nm thick and terminating in a narrow (2 nm) channel, and a terminal anchor-like structure formed by two furled filaments, each 4 nm wide. The virions carry 11 major structural proteins (90, 80, 70, 60, 48, 45, 32, 22, 16, 14 and 12 kDa). The 80 kDa protein appears to be modified in the two-tailed but not in the tail-less virions [1]. High resolution structures of the 32 and 14 kDa proteins have been solved [3, 4] and both display unique folds that are not observed in proteins of other classified viruses [5].

**Table 1.** Characteristics of the family *Bicaudaviridae*

Typical member:	Acidianus two-tailed virus (AJ888457), species *Acidianus two-tailed virus*, genus *Bicaudavirus*	
Virion	Spindle-shaped upon release (120×80 nm), subsequently developing two tails each of up to 400 nm in length	
Genome	Circular dsDNA of 62 730 bp	
Replication	Lytic or lysogenic	
Translation	Not known	
Host range	Hyperthermophilic archaea from the genus *Acidianus*	
Taxonomy	One genus including a single species	

## Genome

**Fig. 2.**
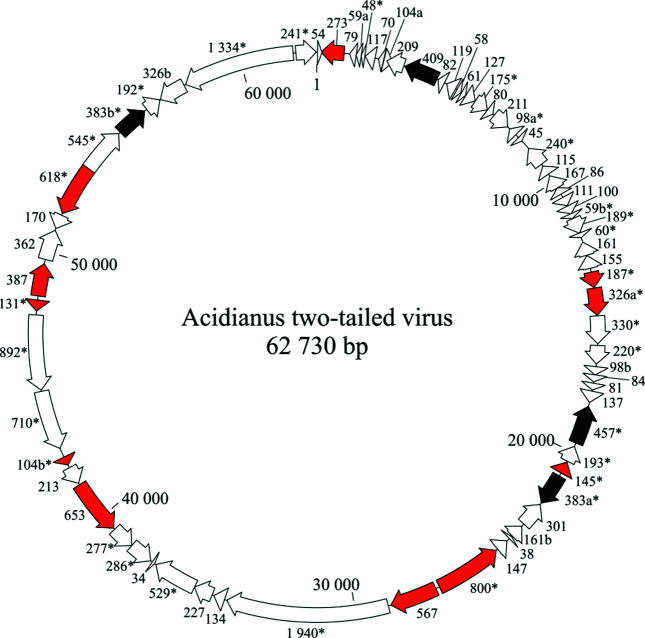
Genome organization of Acidianus two-tailed virus showing putative genes. ORFs are coloured red for virion proteins and black for transposable elements. ORFs shared with unclassified large spindle-shaped viruses are indicated with asterisks.

Virions of Acidianus two-tailed virus contain a circular dsDNA of 62 730 bp (41.2 % GC), one of the largest genomes among crenarchaeal viruses [5], predicted to encode 72 proteins and carry four putative transposable elements (Fig. 2). Forty-three genes are predicted to produce leader-less transcripts and 35 genes are organised into 12 putative operons. Besides structural virion proteins, the virus encodes several enzymes, including a putative integrase of the tyrosine recombinase superfamily, three distinct AAA+ ATPases, a glycosyltransferase, a ParB-like partitioning protein, a DNA repair photolyase and an acyltransferase.

## Replication

Infection leads either to viral replication and subsequent cell lysis or conversion of the infected cell into a lysogen. The lysogenic cycle involves integration of the viral genome into the host chromosome, probably facilitated by the virus-encoded integrase. Lysogeny can be interrupted by stress factors, e.g. UV-irradiation or a decrease in temperature. The virus does not encode identifiable DNA and RNA polymerases and is likely to depend on host cell machinery for genome replication and transcription.

## Taxonomy

Acidianus two-tailed virus was isolated from a hot acidic spring (87–93 °C, pH 1.5–2.0) in Pozzuoli, Italy. The host range is limited to autochthonous species of hyperthermophilic archaea from the genus *Acidianus*. A closely related virus, Acidianus two-tailed virus 2 (KX607101), which shares 90 % identity over 92 % of the genome, is unclassified. Bicaudaviruses are related to a group of unclassified crenarchaeal spindle-shaped viruses (monocaudaviruses) whose virions contain a single long tail that appears to develop intracellularly [6–9]. These viruses share many genes with Acidianus two-tailed virus (Fig. 2) and may represent a new genus within the family *Bicaudaviridae* [10]. Bicaudaviruses encode a conserved DnaA-like AAA+ ATPase, which is shared with members of the archaeal virus families *Fuselloviridae* and *Guttaviridae*, suggesting that the three groups of viruses might be evolutionarily related [11].

## Resources

Full ICTV Online (10th) Report: www.ictv.global/report/bicaudaviridae.
